# The St. Jude Children’s Research Hospital After Completion of Therapy Clinic

**DOI:** 10.1007/s11764-023-01519-6

**Published:** 2024-01-31

**Authors:** Melissa M. Hudson

**Affiliations:** Director, Survivorship Division, St. Jude Children’s Research Hospital, Memphis, TN USA

**Keywords:** Childhood cancer, Survivorship, Long-term follow-up

## Abstract

**Abstract:**

The St. Jude’s After Completion of Therapy (ACT) Clinic was established in 1984 to address the needs of long-term survivors treated at St. Jude Children’s Research Hospital. Survivors eligible for transfer to ACT Clinic include those treated at St. Jude who are cancer-free, 5 years from diagnosis (5 years after completion of relapse therapy), and 2 years after completion of therapy. Services provided to clinic attendees include transportation, housing, and medical care costs not covered by insurance. The clinic’s mission is to improve the quality of life of survivors by facilitating their access to resources that optimize physical and emotional health, social functioning, and educational and vocational achievement. ACT evaluations are undertaken by a multidisciplinary team comprised of nurses, advanced practice providers, physicians, social workers, psychologists, and other medical subspecialists as needed. ACT interventions include the organization of a survivorship care plan/treatment summary, risk-based health screening, counseling about health risks/risk mitigation, comprehensive psychosocial assessment, assistance with care transitions, and case management for identification of local resources. The ACT Clinic offers educational opportunities to graduate medical trainees and precepts national and international visitors seeking guidance in the development of survivorship programs. The ACT Clinic also provides a robust infrastructure for research investigations that have aimed to characterize health outcomes in long-term survivors and test interventions to prevent/remediate adverse effects of childhood cancer and its therapy. Findings from research facilitated by the ACT Clinic have informed health surveillance recommendations for long-term survivors and guided interventions to promote healthy aging among this growing population.

**Implications for Cancer Survivors:**

This review describes a model of care that addresses the medical and psychosocial challenges of survivorship while integrating research investigations to improve health outcomes among childhood cancer survivors.

## Introduction

Inspired by a dream and fund-raising efforts of the late entertainer Danny Thomas and the American Lebanese Syrian Associated Charities (ALSAC), St. Jude Children’s Research Hospital opened its doors in Memphis, TN in 1962 as the first fully integrated children’s hospital in the South. The hospital’s mission focuses on conducting basic and clinical research and developing treatments for childhood cancer, sickle cell disease, and other life-threatening diseases with the vision that no child should die in the dawn of life. The concept of providing care to all children, regardless of a family’s race, religion, or financial status remains integral to the St. Jude culture.

Children are eligible for treatment at St. Jude if they live in Memphis or one of its affiliate program hospitals’ catchment areas; children outside of these regions may also be accepted for treatment if they are eligible for an open clinical trial. Age eligibility for protocols varies by diagnosis, with some protocols enrolling young adults up to age 22 years. Consequently, most patients are from Midsouth and Midwest states, with a sizable proportion representing low-income and medically underserved populations. The cancer patients seen at St. Jude reflect the population-based racial and ethnic demographics of this catchment area and largely include children of Non-Hispanic White race and ethnicity (~70%) followed by Non-Hispanic Black/African American (~20%) and less than 10% Hispanic.

## History of the After Completion of Therapy (ACT) Clinic

**Fig. 1:**
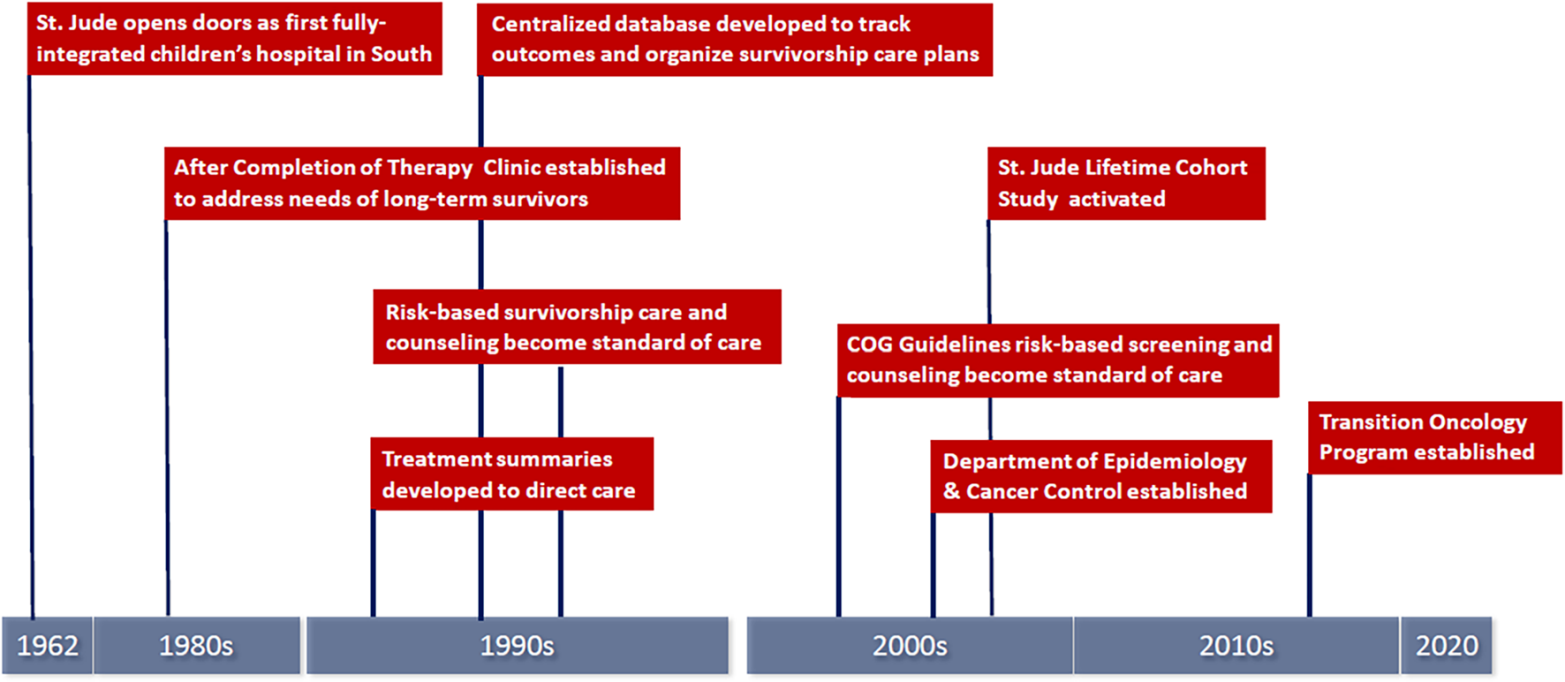
Milestones in survivorship care at St. Jude Children’s Research Hospital’s After Completion of Therapy Clinic

Figure 1 summarizes milestones that have influenced survivorship care at St. Jude. The ACT Clinic was established in 1984 to address the needs of the emerging population of long-term survivors treated at St. Jude. At that time, St. Jude clinicians recognized the benefits of evaluating survivors with sustained remission in an environment distinct from that of acute oncology care. Supported by the philanthropic activities of ALSAC, ACT Clinic services provided to long-term survivors and their families are similar to that of acute oncology care and include funding for transportation, housing, and medical costs not covered by insurance.

The mission of ACT Clinic is to improve the quality of life of survivors by facilitating their access to resources that optimize physical and emotional health, social functioning, and educational and vocational achievement. Survivors eligible for transfer to ACT Clinic include those treated at St. Jude who are cancer-free, 5 years from diagnosis (5 years after completion of relapse therapy), and 2 years after completion of therapy. Survivors treated at other institutions who meet these criteria and move into the Memphis catchment area are also eligible for ACT Clinic services. In recent years, survivors with persistent low-grade neoplasms (e.g., low-grade glioma) have also been transferred to provide them with access to survivorship-focused educational, psychosocial, and transition services. In addition, a shared-care option is available in which the survivor maintains primary affiliation with the oncology care clinic and has access to survivorship services through consultation. Survivors graduate from ACT Clinic services when they are 10 years from diagnosis (or 10 years from completion of relapse therapy) and at least 18 years of age and have graduated from high school. Survivors with cognitive or other special needs participating in community educational and vocational programs are eligible for delayed graduation at 21 years of age. There is no upper age limit for ACT participation, and young adults accepted for cancer treatment are eligible for ACT services until they reach the 10-year diagnosis milestone.

## Evolution to risk-based survivorship care

ACT Clinic interventions have evolved over time. In the early years, survivorship evaluations were limited, still focusing on assuring families of sustained remission. The impact of cancer treatment on physical and intellectual development in young children was increasingly recognized and prompted multidisciplinary collaborations to address clinical needs and characterize survivors at high risk for adverse outcomes who may benefit from surveillance and intervention. In later years, communications from older alumni survivors relating health events and lack of awareness by local providers of well-established associations between specific treatment exposures and late effects stimulated educational efforts to address these deficits. Considerations in care planning at this time included age at diagnosis and targeted treatment exposures like anthracyclines and cranial radiation. Potential late-effects risks associated with protocol therapy were discussed at pre-clinic staff meetings to anticipate the need for evaluations. Eventually, a “risk-based” care approach was implemented that considered specific health risks related to survivor demographics and treatment exposures. A centralized database was developed in 1994 to collect detailed information about cancer diagnosis, chemotherapy agents and doses, radiation treatment fields and doses, and surgical procedures. This database facilitated the development of survivorship care plans (SCPs), initially called ACT Clinical Summaries, which became a standard of care by the mid-1990s. SCP content has evolved over time. In the early years, the plan was limited to a treatment summary, a chronology of major health events, and a list of special concerns related to specific cancer treatments. Exposure-based surveillance recommendations from the Children’s Oncology Group Long-Term Follow-Up Guidelines for Survivors of Childhood, Adolescent, and Young Adult Cancers (COG Guidelines) were incorporated into care plans in 2004 and updated with guideline version updates [1]. Currently, SCPs include cancer diagnostic information, a detailed treatment summary, a list of key health events occurring during and after completion of therapy, a summary of cancer and treatment-related health risks, and exposure-based screening recommendations as well as information about immunizations and family history.

## Data systems supporting survivorship care

In addition to the centralized survivorship database, the institutional health information system has served as a consistent source of downloadable information about survivor demographics and cancer diagnosis for SCPs. Research protocol databases have also been leveraged for cumulative doses of specific chemotherapy agents and radiation fields. The first electronic medical record system at St. Jude was implemented in 2003. While the functionality of these systems has improved over time, the development of SCPs still requires retrospective medical record review to assure comprehensive ascertainment of treatment exposures and health events, to identify cancer treatment interventions undertaken at other institutions, and to curate outcomes into concise and comprehensive documents. Historically, research associates in the Division of Cancer Survivorship performed medical record abstractions to develop treatment summaries and SCPs. These activities moved to the Department of Epidemiology and Cancer Control in 2005 to facilitate the concurrent development of data systems used in research analyses. SCPs remain essential to educational efforts targeting survivors and families and communication with providers involved in survivorship care. SCPs are updated after every ACT Clinic visit and posted on the patient portal of the electronic medical record.

## Clinical care delivery: components of ACT clinic evaluations

ACT evaluations are undertaken by a multidisciplinary team comprised of nurses, advanced practice providers (APPs), physicians, and social workers. Designated liaisons from the Department of Psychology and School Program facilitate and provide consultations as needed. ACT evaluations start with the completion of a questionnaire assessing a review of symptoms, interval health evaluations and hospitalizations, chronic medication use, living/marital status, academic level/grades, and health insurance access. Adolescent and young adult survivors are asked to self-report health behaviors related to sun protection, sedentariness, tobacco, alcohol, cannabis and illicit drug use, sexual activity, and perceptions of suicidality. Adult survivors are also asked to respond to validated health-related quality-of-life measures such as pain, sleep quality, depression, and anxiety. The interventions provided by the multidisciplinary ACT team are summarized in Table 1. In addition, a variety of medical subspecialty (e.g., endocrinology, neurology, cardiology), psychosocial (e.g., psychology, school program), and rehabilitation services (e.g., physical therapy, audiology) are available on campus or at nearby facilities to assist with evaluation, management, and referral for cancer-/treatment-related health conditions identified by the ACT team.

**Table 1:** Interventions provided by the multidisciplinary ACT clinic team

ACT team member	Roles and responsibilities in supporting survivorship care and research
Research associates	– Perform medical record abstractions to develop survivorship care plans – Update health events communicated by survivors or identified during clinical evaluations on survivorship care plans – Codify and severity grade clinical events for research analyses
Nurse	– Triage survivor communications – Provide ACT introduction about clinic operations and interventions for newly transferred survivors – Distribute questionnaires during annual evaluations – Obtain releases for medical and immunization records
Advanced practice providers	– Perform pre-clinic review of interval communications and scheduled evaluations – Review responses to ACT questionnaires with survivors and families – Perform comprehensive clinical examinations – Explain the results of clinical evaluations – Counsel about cancer- and treatment-related health risks and measures for risk mitigation – Assist in the coordination of/transition to community care – Precept visitors and trainees attending the clinic – Document health events for survivorship care plan updates and research analyses
Physicians	– Serve as “doc of the day” and late effects content expert during clinic sessions – Precept visitors and trainees (pediatric hematology-oncology fellows, medical oncology fellows, pediatric residents, and medical students) rotating in the clinic – Consult with all survivors/families at targeted milestones (ACT Clinic entry and graduation) and as needed based on medical and psychosocial concerns identified by the team to address ongoing health concerns, progress/challenges in organizing local healthcare, and the role of ACT Clinic staff in facilitating access to local medical and psychosocial services and resources – Mentor team in the practice of evidence-based survivorship care – Oversee collection of high-quality codified survivorship outcomes data – Participate in educational programs to increase institutional, national, and global awareness of survivorship health outcomes
Social workers	– Perform comprehensive social work assessments at targeted milestones (ACT Clinic entry, mid-teen years (~15 to 16 years of age), pre-alumnus, ACT graduation visit), and as needed for active psychosocial issues. – Identify psychosocial factors that may affect survivorship including general health and wellness, family and interpersonal relationships, insurance and medical care access, educational progress, and vocational plans – Provide education, resources, and referrals to optimize survivorship outcomes
Psychologists	– Perform psychological consultations on survivors identified by the ACT team to have mental health and other psychological challenges – Perform neuropsychological evaluations on survivors identified by the ACT team to have educational/vocational challenges

## Educational opportunities offered by the ACT Clinic

The ACT Clinic has a strong educational mission that includes local/regional, national, and international efforts. Pediatric hematology-oncology fellows from St. Jude rotate annually through the clinic as do medical oncology fellows from the University of Tennessee Health Science Center (UTHSC). In addition, UTHSC pediatric and medicine-pediatric residents have elective options to attend ACT Clinic. The clinic also staff precepts visitors from the US and international pediatric cancer centers seeking guidance in the development of survivorship programs. Finally, a 1-year post-doctoral fellowship is available for individuals interested in training to enhance skills in clinical and research topics focused on survivorship outcomes.

## Trends in ACT Clinic utilization and staffing

Transfer to ACT Clinic is at the discretion of the primary oncology attending and has increased over the years with awareness of the educational and psychosocial services offered by the clinic. Historically, specific diagnostic (e.g., CNS tumor) or treatment (e.g., hematopoietic cell transplant) groups have been retained in acute oncology care beyond 5-year survival; data from recent years indicate completion of ACT evaluations by close to 90% of eligible survivors. Currently, the number of 5-year cancer survivors followed at St. Jude exceeds 8000. Among these, approximately 3700 cancer survivors under 18 years of age who are 5- to 10-years from cancer diagnosis are under active ACT Clinic care. Since 2010 (excluding the COVID-19 pandemic years), annual transfers from the treating oncology clinics into ACT Clinic averaged 286, and annual graduations have ranged between 150 and 200; post-pandemic transfers exceeded 400 in 2022 due to a backlog of newly transferred survivors. After graduation, the St. Jude Cancer Registry monitors cancer and vital status on a regular basis and supplements follow-up data with National Death Index searches.

Staffing of ACT Clinic services has increased with the growth of the survivor population and currently includes six pediatric oncologists who share responsibility for “doc of the day” (three of whom are trained in pediatrics and internal medicine, and two of whom maintain active oncology practices), one full-time staff physician (family practice), eight APPs, two nurses, five social workers, and two psychologists. Research associates in the Department of Epidemiology and Cancer Control support survivorship clinic efforts by developing and updating SCPs that are distributed to survivors after visits with instructions to share with local healthcare providers. Clinic metrics that are routinely tracked include clinic attendance, with specific attention to identifying survivors who fail to transition after transfer from acute oncology care and established ACT survivors who fail to show up for appointments. Previous research by the ACT team identified demographic, medical, and logistic barriers to clinic attendance that remain relevant today [2]. Recent quality improvement projects in ACT have focused on improving adherence to recommended immunizations (e.g., human papilloma virus vaccination for cancer prevention), identifying survivors with loss of vaccine-related immunity (e.g., measles, hepatitis B), and supporting transition to primary care (e.g., identifying primary care provider).

## St. Jude survivorship research

The ACT Clinic also provides a robust infrastructure for research investigations that have aimed to characterize health outcomes in long-term survivors and test interventions to prevent/remediate adverse effects of childhood cancer and its therapy. Undoubtedly, the St. Jude Lifetime Cohort Study, initiated in 2007, represents the most impactful of the ACT-supported research initiatives [3]. SJLIFE is an observational clinical research study that aims to facilitate longitudinal clinical evaluation of health outcomes in aging adults surviving pediatric cancer. SJLIFE evaluations take place in the ACT Clinic and other clinical service areas and research laboratories within St. Jude. To date, over 6200 survivors have been enrolled in SJLIFE and over 5500 survivors have completed one or more comprehensive clinical assessments. The infrastructure of SJLIFE has provided the platform for 51 externally funded projects, including multiple intervention trials, and the development of a Survivorship Portal on the St. Jude Cloud. In addition, SJLIFE research has been featured in over 230 publications since 2011; 40 documenting genetic or epigenetic contributions to treatment-associated adverse long-term outcomes; 11 contributing to risk prediction models for adverse outcomes like heart failure, stroke, and ischemic heart disease [4, 5]; 36 providing support for the COG Guidelines, version 6; and 72 providing support for the NCI Physician Data Query (PDQ). Clinical, genetic, and some physiologic data have also provided strong evidence of multimorbidity associated with the childhood cancer survivor experience [6, 7] and a premature or accelerated aging phenotype characterized by the frailty phenotype and biomarkers of aging such as reduced telomere length, epigenetic alterations, and genomic instability [8–11]. These findings have informed multiple external funding investigations aiming to evaluate interventions to support healthy aging in survivors focused on preserving cardiovascular and brain function.

## Challenges and future opportunities

Through unparalleled institutional support, the ACT Clinic has been able to provide exceptional clinical resources and an ideal platform for observational studies and health promotion intervention trials focused on advancing knowledge and mitigating the late effects of childhood cancer. Despite these advantages, survivors and families report challenges and unmet needs when they transition from St. Jude care to local primary care. The St. Jude Transition Oncology Program (TOP) was organized in 2018 to integrate transition support during acute oncology care with the goal of strengthening relationships with local healthcare providers. Because TOP focuses on newly diagnosed patients and is relatively early in program implementation, its impact on transition outcomes has not been established. Evaluating the impact of TOP services on transition readiness/success as these survivors enter ACT is a priority, as well as enhancing educational and transition services for established ACT survivors. Concurrent with goals to support patient-centered transition programs are research initiatives investigating both survivor and healthcare provider perspectives about preferred resources and platforms to facilitate survivorship care. In these efforts, years of partnership with survivors and families have underscored the importance of maintaining a strong clinical infrastructure that prioritizes survivors’ needs as we address knowledge gaps about survivorship care and outcomes.

## References

[1] Landier W, Bhatia S, Eshelman DA (2004). Development of risk-based guidelines for pediatric cancer survivors: the children’s oncology group long-term follow-up guidelines from the children’s oncology group late effects committee and nursing discipline. J Clin Oncol..

[2] Klosky JL, Cash DK, Buscemi J (2008). Factors influencing long-term follow-up clinic attendance among survivors of childhood cancer. J Cancer Surviv..

[3] Howell CR, Bjornard KL, Ness KK (2021). Cohort profile: the St. Jude lifetime cohort study (SJLIFE) for paediatric cancer survivors. Int J Epidemiol..

[4] Chow EJ, Chen Y, Kremer LC (2015). Individual prediction of heart failure among childhood cancer survivors. J Clin Oncol..

[5] Chow EJ, Chen Y, Hudson MM (2018). Prediction of ischemic heart disease and stroke in survivors of childhood cancer. J Clin Oncol..

[6] Hudson MM, Ness KK, Gurney JG (2013). Clinical ascertainment of health outcomes among adults treated for childhood cancer. JAMA..

[7] Bhakta N, Liu Q, Ness KK (2017). The cumulative burden of surviving childhood cancer: an initial report from the St Jude lifetime cohort study (SJLIFE). Lancet..

[8] Song N, Li Z, Qin N (2020). Shortened leukocyte telomere length associates with an increased prevalence of chronic health conditions among survivors of childhood cancer: a report from the St. Jude lifetime cohort. Clin Cancer Res..

[9] Qin N, Li Z, Song N (2021). Epigenetic age acceleration and chronic health conditions among adult survivors of childhood cancer. J Natl Cancer Inst..

[10] Hagiwara K, Natarajan S, Wang Z (2023). Dynamics of age- versus therapy-related clonal hematopoiesis in long-term survivors of pediatric cancer. Cancer Discov..

[11] Delaney A, Howell CR, Krull KR (2021). Progression of frailty in survivors of childhood cancer: a St. Jude lifetime cohort report. J Natl Cancer Inst..

